# SRCAP is involved in porcine reproductive and respiratory syndrome virus activated Notch signaling pathway

**DOI:** 10.1128/jvi.01216-24

**Published:** 2024-11-12

**Authors:** Guofei Ding, Yingchao Li, Dexin Li, Mingyu Dou, Chaolun Fu, Ting Chen, Xinyu Cui, Qin Zhang, Pingping Yang, Yanmeng Hou, Sidang Liu, Yihong Xiao

**Affiliations:** 1 Department of Fundamental Veterinary Medicine, College of Veterinary Medicine, Shandong Agricultural University, Tai’an, Shandong, China; 2 Shandong Provincial Key Laboratory of Animal Biotechnology and Disease Control and Prevention, Shandong Agricultural University, Tai’an, Shandong, China; University of Michigan Medical School, Ann Arbor, Michigan, USA

**Keywords:** porcine reproductive and respiratory syndrome virus, non-structural protein 4, interactions, SRCAP, non-canonical Notch signaling, antiviral drug

## Abstract

Porcine reproductive and respiratory syndrome viru*s* (PRRSV) is the cause of porcine reproductive and respiratory syndrome (PRRS); a disease of pigs, which results in great economic losses in the pork industry. The non-structural protein 4 (Nsp4), a 3C-like serine protease responsible for most non-structural protein processing, plays an essential role in PRRSV infection. We used label-free quantitative proteomics to elucidate the Nsp4 interactome and SRCAP was identified as one of the interactors. SRCAP facilitated PRRSV infection by activating non-canonical Notch signaling. The ATPase I–IV domain in SRCAP and the ^122^VITEA^126^ in Nsp4 were identified as the interacting sites. The infection of recovered mutant rTA-12/5A (^122^AAAAA^126^) could not activate Notch signaling. The results indicated that ^122^VITEA^126^ in Nsp4 were key sites to determine the function of SRCAP and their interaction. A function of Nsp4 in activating the Notch signaling pathway was discovered. Block Notch signaling pathway could inhibit PRRSV infection both *in vitro* and *in vivo* which may lead to the development of novel therapeutic antiviral strategies.

**IMPORTANCE:**

In the present study, the interactome of the NSP4 originating from PRRSV was studied and SRCAP was confirmed as one of the interactors. Mechanism study showed the interaction of Nsp4 and SRCAP was found to facilitate PRRSV infection by activating non-canonical Notch signaling. ATPase Ⅰ–Ⅳ domain in SRCAP and the ^122^VITEA^126^ in Nsp4 were identified as the interacting sites that demined the activating of Notch signaling. Block Notch signaling pathway could inhibit PRRSV infection *in vitro* and *in vivo* which may be a new target for antiviral drug development.

## INTRODUCTION

Porcine reproductive and respiratory syndrome virus (PRRSV) is the cause of porcine reproductive and respiratory syndrome (PRRS); a disease of pigs, which results in great economic losses in the pork industry. Symptoms of PRRS include reproductive failure in pregnant sows and respiratory distress in pigs of all ages. This disease was first reported in North America in 1989 (1), and the causative virus, PRRSV, was isolated in 1991 (2). In China, reports of PRRSV-infected pigs first emerged in 1995; a decade later, a severe outbreak occurred throughout China that was subsequently found to be caused by a mutated PRRSV: the highly pathogenic PRRSV (HP-PRRSV) where a 90-bp deletion had occurred in the genome that encoded for the non-structural protein (Nsp) 2 (3, 4). In 2014, the variant PRRSV NADC30-like strain, a 393-bp deletion had occurred in the Nsp2 gene, similar to the NADC30 strain that was first isolated in the United States (in 2008) was reported in China (5). Increasingly, there are reports of a variety of new PRRSV strains emerging, indicating that PRRS is an ongoing threat to the swine industry worldwide (6, 7).

The Notch signaling pathway is evolutionary conserved through direct cell-to-cell communication between neighboring cells which plays a critical role in many fundamental processes, including cell proliferation, apoptosis, activation of differentiation programs, and specific cell fates (8). It can be initiated by Notch receptor−ligand interaction and subsequent enzymatic proteolysis. The γ-secretase catalyzes the terminal cleavage event (S3 cleavage), which releases a fragment known as the Notch intracellular domain (NICD). The NICD fragment then translocates to the nucleus to initiate the downstream transcriptional machinery (8, 9). Virus-related diseases, including PRRS, always alters the normal function of the Notch signaling (10–12). Many drugs with Notch-targeting therapeutic strategies have been searched for and are in the preclinical stage or tested in clinical trials, especially in treating cancer, immunity, and inflammatory disorders (9). The Snf-2-related CREB-binding protein activator protein (SRCAP) is a 350-kDa protein that belongs to the SNF-2 family of ATPases, the members of which function in regulating transcription and remodeling chromatin (13, 14). SRCAP has been shown to bind to CBP and functions as a coactivator to regulate gene transcription, including genes involved in the Notch signaling pathway (15). SRCAP has been reported to function as a transcription coactivator in the Notch signaling pathway (16, 17). Several viruses have been reported to interact with SRCAP to regulate the infection (15, 18, 19). SRCAP was identified as a candidate for the antiviral activity against RSV by acting as a scaffold protein (20).

PRRSV is an enveloped, single-stranded, positive-sense RNA virus that was recently reassigned to the order: *Nidovirales*; family: *Arteriviridae*; and genus: *Porarteviru*s. The genome is approximately 15 kb in size with a 5′ cap and 3′ polyadenylate tail; it contains at least 10 open reading frames that encode 8 structural proteins (GP2, E, GP3, GP4, GP5, GP5a, M, and N) (21, 22); and 14 Nsps produced by auto-proteolytic cleavage of Nsp2 and Nsp4 (23). Nsp4 is a protease generated by the cleavage of precursor polyprotein 1a, a serine protease that shares properties with chymotrypsin-like enzymes, such as 3C-like serine proteases; and is responsible for the processing of Nsp3–Nsp12; all of which play key roles in both the life cycle and virulence of the PRRSV (24, 25). Given Nsp4’s core role in PRRSV life cycle, we hypothesized that cellular proteins interacting with Nsp4 may contribute to its proteolytic cleavage activity. Therefore, these cellular proteins could be potential therapeutic targets. Here, label-free quantitative proteomics was used to elucidate the Nsp4 interactome. SRCAP was found as an interactor with high possibility. In this study, the role of SRCAP in PRRSV infection and Notch signaling pathway was discovered.

## RESULTS

### Expression of EGFP-Nsp4 and interaction analysis

**Fig 1:**
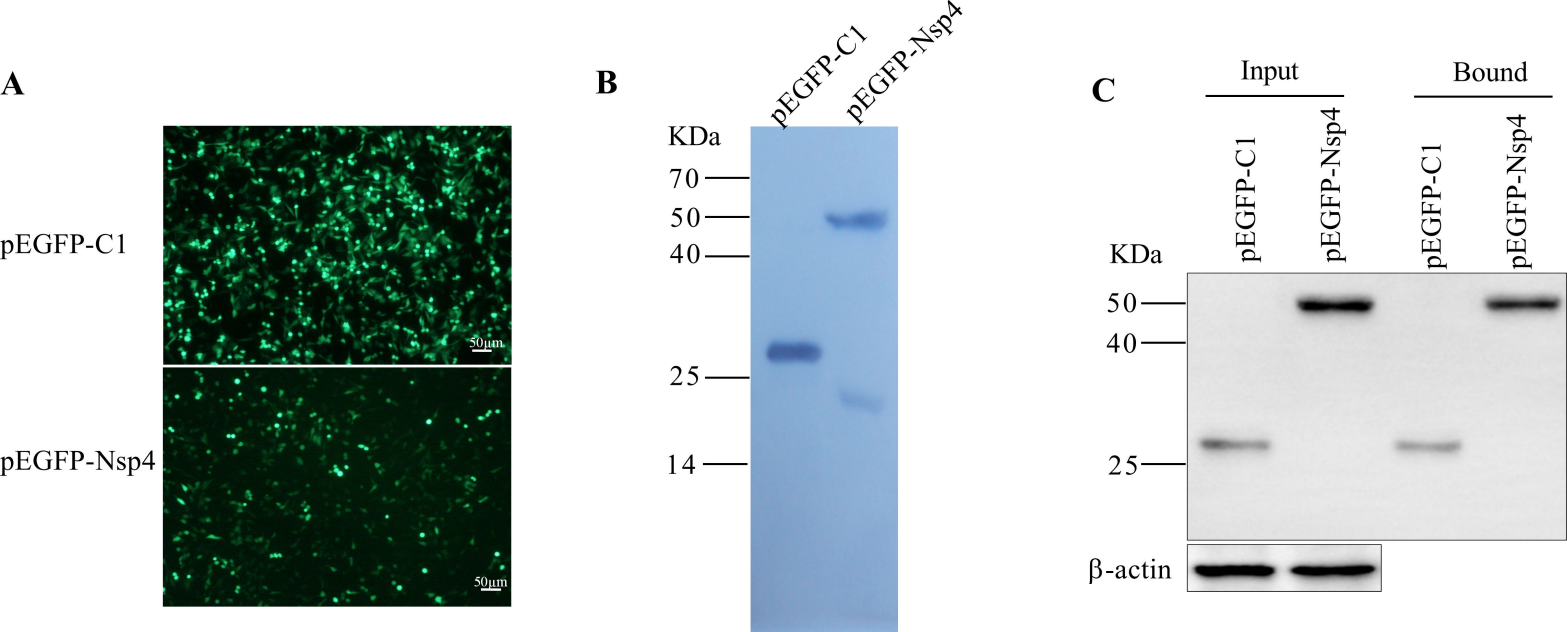
Analysis of the Nsp4 interactome Expression of GFP and GFP-Nsp4. The recombinant plasmid pEGFP-Nsp4 and vector were transfected into 293T cells. Twenty-four hours later, the cells were fixed and validated by fluorescence microscopy (**A**), or collected and the whole-cell lysate was prepared for Western blot (**B**) analysis. The predicted molecular sizes were 26.9 and 55 kDa, respectively. (**C**) GFP and GFP-Nsp4 were detected by Western blot. Nsp4 expression was verified in pEGFP-Nsp4 and vector-transfected 293T cells using GFP-trap pull-down and by Western blot.

To determine the interactome of the PRRSV Nsp4 protein, a combined EGFP-trap LC-MS/MS approach was used, similar to the approach we have used to elucidate the interactome of PRRSV Nsp2 (26). The expression of Nsp4 was confirmed in 293T cells both direct observation under fluorescence microscopy (Fig. 1A) and Western blot (Fig. 1B). Cellular proteins interacting with Nsp4 were pulled down by a highly specific GFP-trap which could selectively precipitate EGFP and EGFP fusion proteins (Fig. 1C). The pull-downs were analyzed with label-free LC-MS/MS which was a reliable method for identifying and quantifying the selectively enriched proteins.

To provide a statistically robust data set, pull-downs with both the EGFP control and EGFP-Nsp4 were performed independently in triplicate. The abundance of bound peptides from the EGFP and pEGFP-Nsp4 traps were compared using MaxQuant software against a human database. The assignment of proteins that bound to pEGFP compared to proteins that bound to pEGFP-Nsp4 was quantified by label-free proteomics. The selection criteria for potential interacting partners for Nsp4 were as follows: (1) a false discovery rate ≤1%; (2) two or more unique peptides for reporting protein identifications; (3) a binding ratio greater than twofold. In total, 44 cellular proteins were found that interacted with Nsp4 (Table 1).

**TABLE 1:** Cellular proteins showing more than a twofold change in abundance compared with GFP-NSP4 and GFP

Protein ID	Protein name	Unique peptides	Sequence coverage (%)	Mol. weight (kDa)	Max fold change	*T* test	Protein function(s)
NSP4	NSP4	18	74.5	21.07	7,957.66	0.017	NSP4
Q6ZRS2	SRCAP	2	0.5	343.55	20.81	0.007	Snf2-related CREBBP activator protein which transcriptionally regulate genes by chromatin remodeling
Q14157	UBAP2L	20	28.9	114.53	19.59	0.028	Ubiquitin-associated protein 2-like
P35580	MYH10	72	45.3	229.00	13.67	0.012	Myosin, heavy chain 10, non-muscle
P61224	RAP1B	9	44	20.83	12.76	0.044	RAP1B, member of RAS oncogene family
Q59EG8	PSMD2	13	15.2	100.59	12.53	0.040	Proteasome (prosome, macropain) 26S subunit, non-ATPase, 2
P62633	CNBP	7	41.8	19.46	10.50	0.024	CCHC-type zinc finger, nucleic acid binding protein
Q9P2J5	LARS	7	7.5	134.46	9.55	0.020	Leucyl-tRNA synthetase; catalyzes the specific attachment of an amino acid to its cognate tRNA in a two-step reaction
B0QY89	EIF3L	9	17.1	70.90	9.17	0.045	Eukaryotic translation initiation factor 3, subunit L
Q4LE36	ACLY	12	11.3	124.56	9.00	0.040	ATP citrate lyase; ATP citrate-lyase is the primary enzyme responsible for the synthesis of cytosolic acetyl-CoA in many tissues
D3DQ69	SERBP1	2	64.4	49.09	8.95	0.014	mRNA-binding protein 1
Q14011	CIRBP	6	36.6	18.65	8.73	0.033	Cold inducible RNA-binding protein
P67809	YBX1	17	73.5	35.92	7.96	0.045	Y box-binding protein 1; mediates pre-mRNA alternative splicing regulation; binds and stabilizes cytoplasmic mRNA
Q9Y2S7	POLDIP2	6	18.5	42.03	7.33	0.026	Polymerase delta-interacting protein 2
O60869	EDF1	17	72.3	16.37	6.94	0.037	Endothelial differentiation-related factor 1; transcriptional coactivator stimulating NR5A1 and ligand-dependent NR1H3/LXRA and PPARG transcriptional activities
Q04837	SSBP1	5	39.2	17.26	6.67	0.030	Single-stranded DNA binding protein 1, mitochondrial. This protein binds preferentially and cooperatively to ss-DNA
O14818	PSMA7	9	33.5	27.89	6.50	0.033	Proteasome (prosome, macropain) subunit, alpha type, 7; plays an important role in the regulation of cell proliferation or cell cycle control, transcriptional regulation, immune and stress response, cell differentiation, and apoptosis
A0A024R895	SET	7	30.7	32.10	6.33	0.038	SET translocation (Myeloid leukemia-associated), isoform CRA_b
Q6PKG0	LARP1	30	37.2	123.51	6.18	0.013	La ribonucleoprotein domain family, member 1
Q59FF0	SND1	26	30.7	107.43	5.80	0.049	Staphylococcal nuclease and tudor domain containing 1
P13797	PLS3	21	38.9	70.81	5.40	0.050	Plastin 3; actin-bundling protein found in intestinal microvilli, hair cell stereocilia, and fibroblast filopodia
P23396	RPS3	17	70.8	26.69	5.06	0.032	Ribosomal protein S3
P35268	RPL22	5	51.6	14.79	5.04	0.035	Ribosomal protein L22
P30153	PPP2R1A	9	17.3	65.31	4.83	0.039	Protein phosphatase 2, regulatory subunit A, alpha
P11177	PDHB	8	28.1	39.23	4.66	0.004	Pyruvate dehydrogenase (lipoamide) beta
B4DRM3	EIF4B	25	36.9	69.73	4.58	0.017	Eukaryotic translation initiation factor 4B; required for the binding of mRNA to ribosomes
P62753	RPS6	11	34.9	28.68	4.43	0.030	Ribosomal protein S6
P34932	HSPA4	24	38.1	94.33	4.39	0.044	Heat shock 70 kDa protein 4
P37802	TAGLN2	3	17.1	22.39	4.32	0.038	Transgelin 2
Q15393	SF3B3	11	10.4	135.58	4.23	0.036	Splicing factor 3b, subunit 3
Q06830	PRDX1	9	63.8	22.11	4.21	0.023	Peroxiredoxin 1; involved in redox regulation of the cell
P52815	MRPL12	6	23.7	21.35	4.19	0.033	Mitochondrial ribosomal protein L12
Q13263	TRIM28	21	33.4	88.55	4.17	0.039	Tripartite motif containing 28; transcription intermediary factor 1-beta
P07900	HSP90AA1	13	35	84.66	3.89	0.025	Heat shock protein 90 kDa alpha (cytosolic), class A member 1
P31946	YWHAB	5	48	28.08	3.70	0.048	Tyrosine 3-monooxygenase/tryptophan 5-monooxygenase activation protein, beta polypeptide
Q15181	PPA1	8	30.8	32.66	3.61	0.019	Pyrophosphatase (inorganic) 1
B5MDF5	RAN	7	27.9	26.22	3.55	0.038	RAN, member RAS oncogene family
O75533	SF3B1	10	10.1	145.83	3.46	0.036	Splicing factor 3b, subunit 1; subunit of the splicing factor SF3B required for “A” complex assembly formed by the stable binding of U2 snRNP to the branchpoint sequence (BPS) in pre-mRNA
O43242	PSMD3	7	16.3	60.98	3.37	0.028	Proteasome (prosome, macropain) 26S subunit, non-ATPase, 3
A0A024RDF4	HNRPD	6	54.6	32.83	3.26	0.030	Heterogeneous nuclear ribonucleoprotein D (AU-rich element RNA binding protein 1, 37 kDa), isoform CRA e
P56192	MARS	10	13.3	101.11	3.21	0.017	Methionyl-tRNA synthetase
P05455	SSB	17	31.9	46.84	3.19	0.031	Sjogren syndrome antigen B (autoantigen La); binds to the 3′ poly (U) termini of nascent RNA polymerase III transcripts, protecting them from exonuclease digestion and facilitating their folding and maturation
P13647	KRT5	5	51.9	62.38	2.84	0.026	Keratin 5
P05204	HMGN2	3	54.4	9.39	2.45	0.036	High mobility group nucleosomal binding domain 2; binds to the inner side of the nucleosomal DNA thus altering the interaction between DNA and the histone octamer
P49458	SRP9	7	62.8	10.11	2.31	0.045	Signal recognition particle 9 kDa

### Validation of the Nsp4 interactome

**Fig 2:**
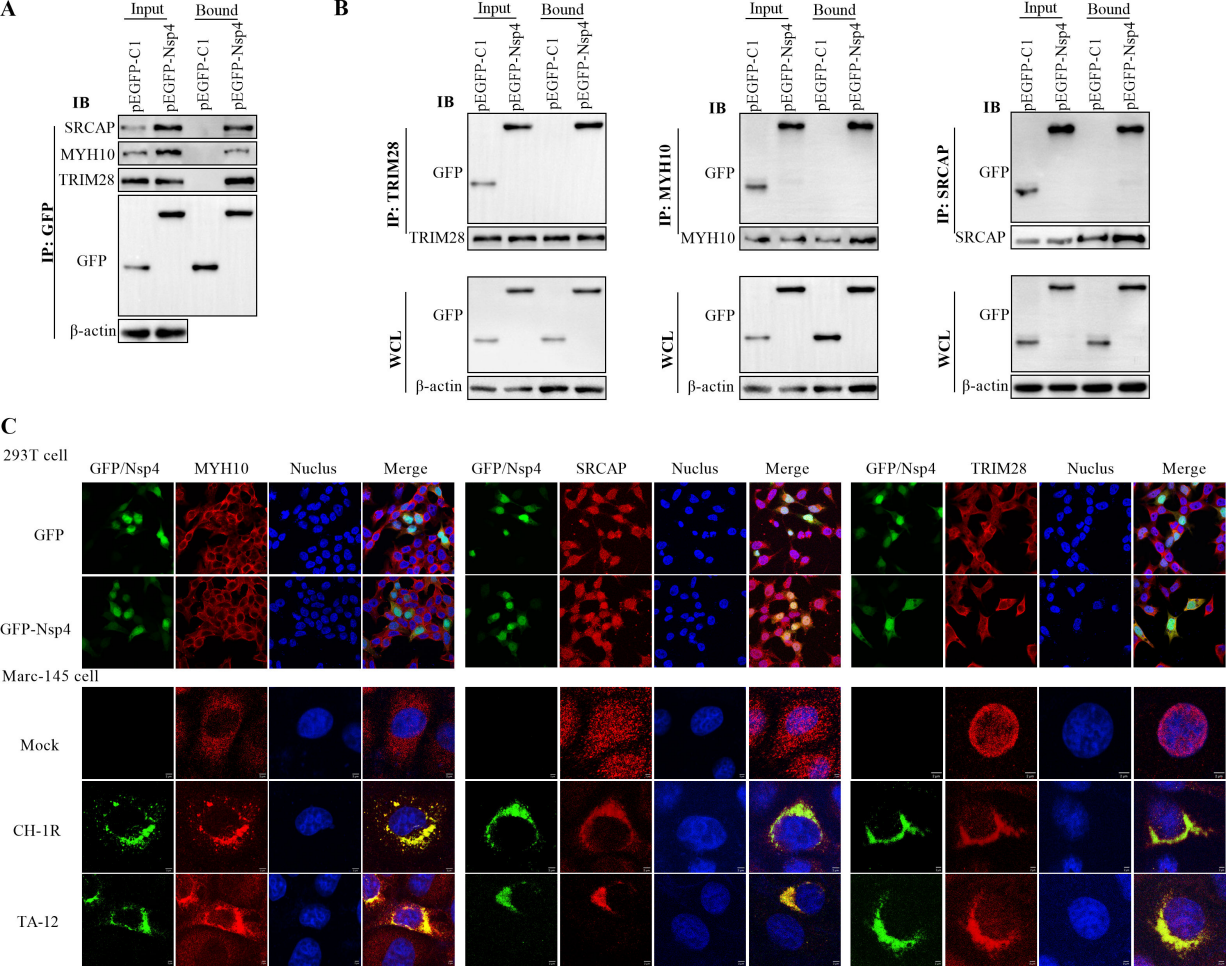
Validation of the GFP-Nsp4 interactome. **(A)** Validation of the interaction of Nsp4 with cellular proteins by pull-down. Constructs of pEGFP-Nsp4 and pEGFP-C1 were transfected HEK293T cells for 24 h, respectively. The Nsp4 interactors were pulled down by GFP-trap and identified by Western blot using specific antibodies against SRCAP, TRIM28, or MYH10. **(B)** Confirmation of the proteins associated with Nsp4 by IP. Blank vector and pEGFP-Nsp4 were transfected HEK293T cells for 24 h. The cell lysates were incubated with specific antibodies against SRCAP, TRIM28, or MYH10 after pre-clearing with blank protein G resin, respectively. The existence of GFP and pEGFP-Nsp4 in the input samples and the eluates were detected by a specific antibody against GFP. **(C)** Co-localization of Nsp4 with cellular proteins confirmed by immunofluorescence assay. The recombinant plasmid pEGFP-Nsp4 and vector were transfected into HEK293T cells. MARC-145 cells were either mock infected or infected with both LP-PRRSV CH-1R strain and HP-PRRSV TA-12 strain at an MOI of 0.01. Twenty-four hours later, the cells were fixed and probed by anti-SRCAP, TRIM28, or MYH10 antibodies, then visualized with Cy3-goat anti-mouse IgG. In MARC-145 cells, Nsp4 was probed with by multi-clonal anti-Nsp4 antibody and visualized by a FITC-goat anti-rabbit antibody. The co-localization was determined by the yellow signal in the merged images. WCL: whole-cell lysate.

The LC-MS/MS identified groups of cellular proteins with different biological functions. The reliability of the LC-MS/MS results was verified by biological replicates with pull-down, immunoprecipitation (IP), and immunofluorescence (IF) assays via selection of three proteins (SRCAP, two peptides, 20.81-fold enhanced; MYH10, 72 peptides, 13.67-fold enhanced; and TRIM28, 21 peptides, 4.17-fold enhanced). These proteins were taken as the variability in peptides used for identification, differences in ratio between pEGFP and pEGFP-Nsp4, and biological functions. A repeat pull-down was carried out using a procedure for the preparation of proteomic samples and eluted samples were validated by antibodies to specific proteins using Western blot. The results showed that SRCAP, MYH10, and TRIM28 were detected in the pull-downs (Fig. 2A). To further confirm interactions, IPs were performed, in which antibodies to specific cellular proteins were tested for their ability to precipitate pEGFP-Nsp4. SRCAP, MYH10, and TRIM28 were found to co-localized with Nsp4 (Fig. 2B).

Co-localization of different proteins was an important detection method to determine possible interactions. In this study, the co-localization of Nsp4 and cellular proteins was verified in pEGFP-Nsp4 transfected 293T cells (used for the pull-down) and PRRSV-infected MARC-145 cells. Using confocal microscopy, Nsp4 was found to co-localize with SRCAP and TRIM28, but not with MYH10 and EGFP (Fig. 2C). Using PRRSV-infected MARC-145 cells, the PRRSV was probed with a multiclonal antibody specific to Nsp4 with results indicating that Nsp4 co-localized with MYH10, SRCAP, and TRIM28 (Fig. 2C). All these results showed the high reliability of the MS.

### SRCAP facilitates PRRSV replication

**Fig 3:**
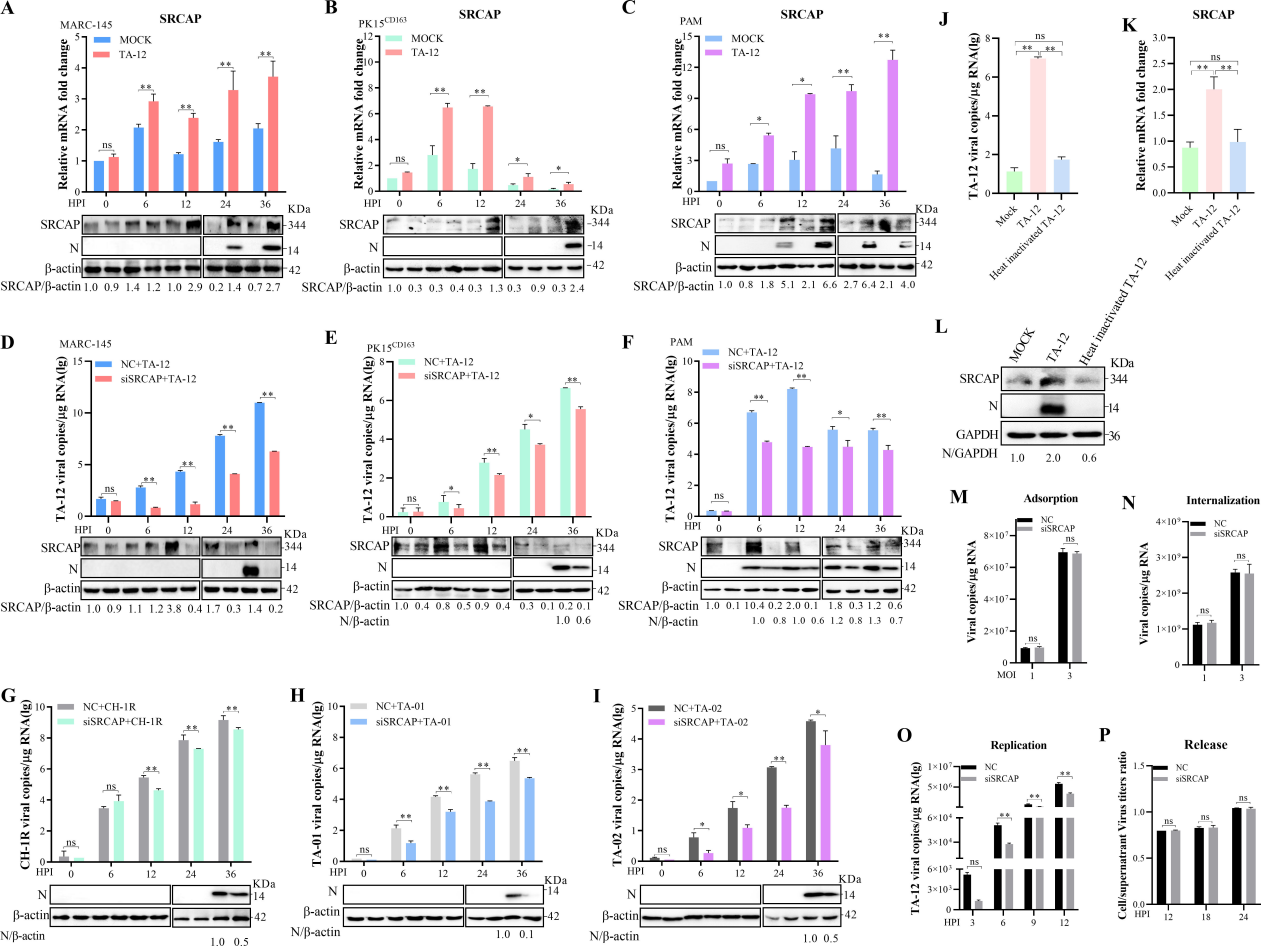
SRCAP facilitated PRRSV infection. **(A through C)** SRCAP expression increased after PRRSV infection. MARC-145 (**A**), PK15^CD163^ (**B**), or PAM (**C**) cells were infected with PRRSV TA-12. Both mRNA and protein were collected at 0, 6, 12, 24, and 36 HPI and detected by qPCR and Western blot, respectively. Bottom: Data were quantified and shown as the ratio of SRCAP to β-actin. **(D through F)** SRCAP facilitated PRRSV infection. MARC-145 (**D**), PK15^CD163^ (**E**), and PAM (**F**) cells were transfected with siRNA targeting SRCAP and non-targeting NC for 24 h and then infected with HP-PRRSV TA-12 virus at an MOI of 0.01. The viral genome, SRCAP, and N proteins were detected by qPCR and Western blot. Bottom: Data were quantified and shown as the ratio of SRCAP and N proteins to β-actin. **(G through I)** SRCAP facilitated different subtypes of PRRSV infection. MARC-145 cells were transfected with siRNA targeting SRCAP and NC for 24 h and then infected with the LP-PRRSV strain (CH-1R) (**G**), NADC30-like strain TA-01 (**H**), or recombinant strain (recombined by HP-PRRSV and NADC30-like) strain TA-02 (**I**). The viral genome and protein were detected by qPCR and Western blot. Bottom: Data were quantified and shown as the ratio of N proteins to β-actin. **(J through L)** No changes of SRCAP level caused by heat-inactivated TA-12. MARC-145 cells were infected with TA-12 and heat-inactivated TA-12. The mock treatment group was established as a control. The viral genome (**J**) and SRCAP mRNA (**K**) were detected by qPCR at 24 h. The SRCAP and N proteins were detected by Western blot (**L**). **(M through P)** Confirming the role of SRCAP in PRRSV life cycle. MARC-145 cells were transfected with siRNA targeting SRCAP and non-targeting NC for 24 h and then infected with HP-PRRSV TA-12 virus at an MOI of 1 and 3, respectively, and absorbed at 4°C for 1 h. After washing with cold PBS, the cells were collected and the viral RNA was extracted. The attached PRRSV particles were evaluated by qPCR to analyse the role of SRCAP in absorption (**M**). The SRCAP knocked down MARC-145 cells infected with HP-PRRSV TA-12 virus at an MOI of 1 and 3, respectively, at 4°C for 1 h and were shifted from 4°C to 37°C for 4 h. After washing with low pH buffer PBS, the cells were collected and the viral RNAs were extracted. The internalized PRRSV particles were evaluated by qPCR (**N**). The SRCAP knocked down MARC-145 cells that were infected with TA-12 virus at an MOI of 0.01. Cells were collected and viral RNA extracted at 3, 6, 9, and 12 h. Genomic PRRSV was evaluated by qPCR (**O**) or the intracellular and extracellular infectious virus particles were determined by the TCID_50_ assay using the freeze-and-thaw cell supernatants and culture supernatants, respectively. The ratio of the extracellular viral titer to the intracellular viral titer indicates the ability to release virus (**P**). Data were quantified and shown as the ratio of SRCAP to β-actin or GAPDH. GAPDH was used as an internal control for qPCR. Error bars indicate the SDs of three experimental replicates. β-actin or GAPDH was used as an internal control for the Western blot. The asterisks indicate a significant difference between the control values: *P < 0.05; **P < 0.01 (unpaired two-tailed Student’s *t* test). HPI: hours post-infection.

As SRCAP interacted with Nsp4 with the highest possibility, the role of the SRCAP in PRRSV infection was detected in MARC-145, PK15^CD163^, and PAM cells. The results showed that the infection of TA-12 could upregulate SRCAP (Fig. 3A through C). Knocking down of SRCAP could decrease the infection of TA-12 (Fig. 3D through F). To confirm the strain specificity, CH-1R, TA-01, and TA-02 were infected into MARC-145 cells. The results showed that the knocking down of SRCAP could decrease the infection of all three types of the PRRSV (Fig. 3G through I). However, the heat-inactivated TA-12 had no influence on the expression of SRCAP (Fig. 3J through L). SRCAP were knocked down in MARC-145 cells to learn the role of SRCAP in viral absorption, internalization, replication, or release. The results showed that knock down of SRCAP could only decrease the replication of the PRRSV (Fig. 3M through P). All these results strongly indicated that SRCAP plays a proviral role during PRRSV replication.

### SRCAP facilitates PRRSV infection by activating non-canonical Notch signaling

**Fig 4:**
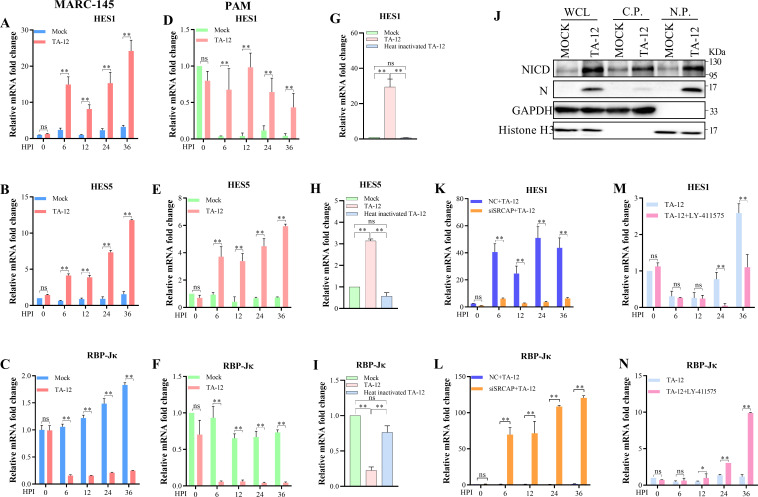
PRRSV activates the non-canonical Notch signaling pathway. **(A through I)** PRRSV induced the expression of Notch signaling-related genes. MARC-145 cells or PAMs were infected with TA-12 at an MOI of 0.01 for 0, 6, 12, 24, and 36 h. The mRNA of HES1 (**A**), HES5 (**B**), and RBP-Jκ (**C**) of MARC-145 cells, HES1 (**D**), HES5 (**E**), and RBP-Jκ (**F**) of PAMs were detected by qPCR. **(G through I)** The level of Notch signaling-related gene expression in heat-inactivated TA-12. MARC-145 cells were inoculated with heat-inactivated TA-12 for 24 h. The mRNA level of HES1 (**G**), HES5(**H**), and RBP-Jκ (**I**) was detected by qPCR.(**J)** PRRSV increased the expression and distribution of NICD. MARC-145 cells were infected with TA-12 for 24 h. WCL, Cytoplasmic protein and Nucleus protein were collected and detected by Western blot. **(K and L)** A knockdown of SRCAP inhibited the TA-12-activated Notch signaling. The siRNA targeting SRCAP was transfected into MARC-145 cells for 24 h, and then infected with TA-12 at an MOI of 0.01 for 0, 6, 12, 24, and 36 h. The level of HES1 (**K**) and RBP-Jκ (**L**) mRNA expression was detected by qPCR. **(M and N)** LY-411575 inhibited the TA-12-activated Notch signaling. MARC-145 cells were pretreated with LY-411575 and then infected with TA-12 for 0, 6, 12, 24, and 36 h. The level of HES1 (**M**) and RBP-Jκ (**N**) mRNA expression was detected by qPCR. GAPDH was used as an internal control for qPCR. The error bars indicate the SDs of three experimental replicates. GAPDH was used as an internal control for Western blot for WCLs and Cytoplasmic protein. Histone H3 was used as an internal control for the Western blot for Nucleus protein. C.P.: cytoplasmic protein; N.P.: nuclear protein. The asterisks indicate a significant difference between the control values: *P < 0.05; **P < 0.01 (Student’s *t* test).

SRCAP has been reported as a coactivator of transcription in Notch signaling (16, 17). So, the Notch signaling in PRRSV infection was confirmed. The results showed that TA-12 infection could upregulate the Notch signaling-related genes, HES1 and HES5, while downregulate RBP-Jκ (Fig. 4A through C). These results were confirmed in TA-12 infected PAMs (Fig. 4D through F). The heat-inactivated TA-12 had no effect on Notch signaling (Fig. 4G through I). The infection of TA-12 could increase NICD and its distribution in the nucleus part (Fig. 4J). All these results showed that TA-12 could activate Notch signaling which required cleavage of the Notch receptor.

To confirm the role of SRCAP in PRRSV-induced Notch signaling, SRCAP was knocked down by specific siRNA. The knocking down of SRCAP could block TA-12 infection, downregulate HES1 and upregulate RBP-Jκ (Fig. 4K and L), which was consistent with those of the LY-411575, an inhibitor of Notch signaling (Fig. 4M and N). Based on PRRSV-activated Notch signaling was RBP-Jκ-independent, it was concluded that PRRSV infection activated non-canonical Notch signaling.

**Fig 5:**
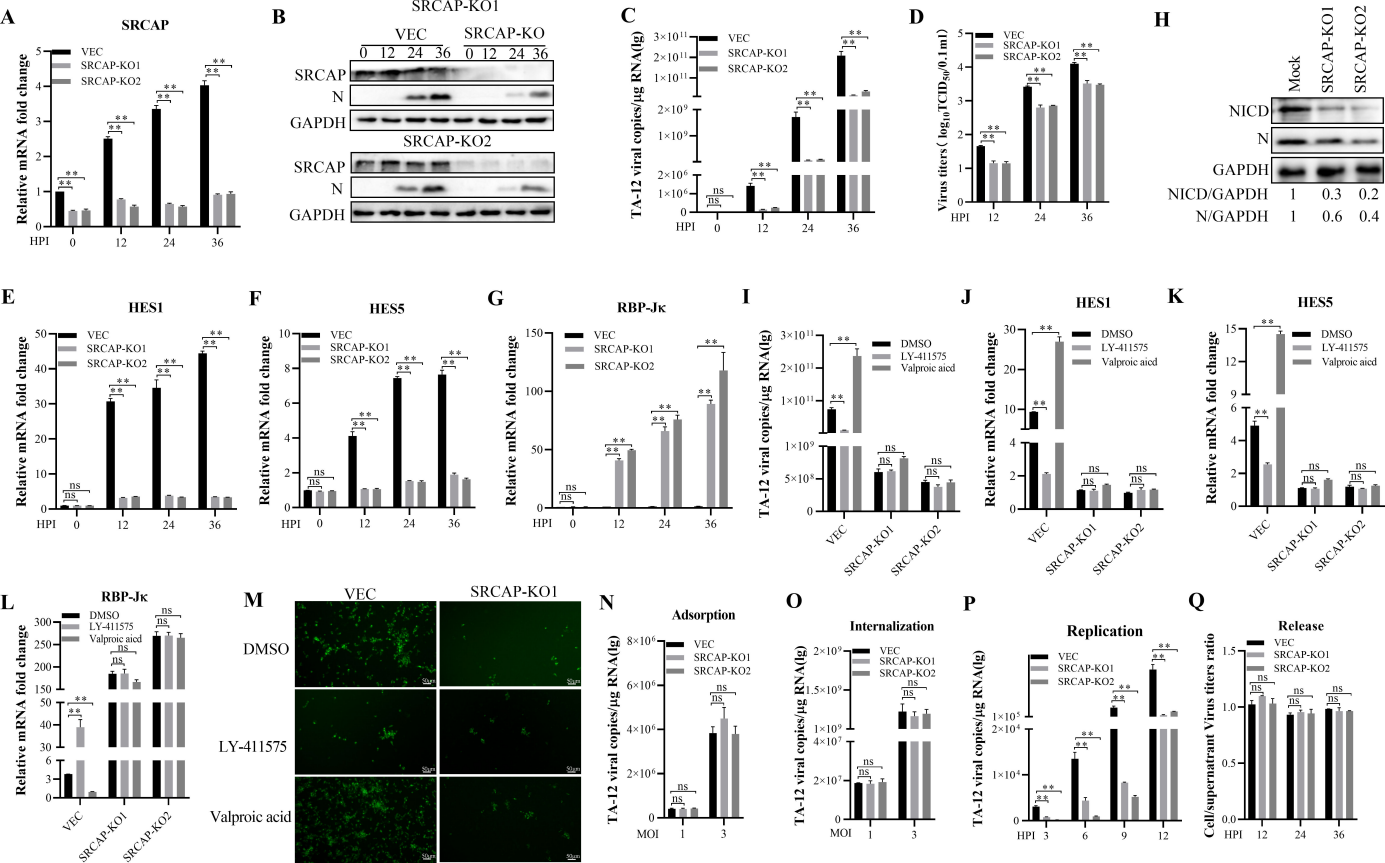
Confirmation of the role of SRCAP in SRCAP-knockout MARC-145 cells. **(A and B)** Construction of SRCAP-knockout MARC-145 cell line. The SRCAP-knockout MARC-145 cell lines SRCAP-KO1 and SRCAP-KO2 were confirmed by qPCR (**A**) and Western blot (**B**). **(B through D)** PRRSV replication was blocked in SRCAP-knockout MARC-145 cell lines. SRCAP-KO1, SRCAP-KO2, and their corresponding control cell line VEC were infected by TA-12 with an MOI of 0.01 for 0, 12, 24, and 36 h. The viral protein was measured by Western blot (**B**), viral genome was detected by qPCR (**C**), and viral titer was titrated (**D**). **(E through G)** Notch signaling pathway was inhibited in SRCAP-knockout MARC-145 cell lines after PRRSV infection. SRCAP-KO1, SRCAP-KO2, and VEC cells were infected with TA-12 at an MOI of 0.01 for 0, 12, 24, and 36 h. The mRNA level of HES1 (**E**), HES5 (**F**), and RBP-Jκ (**G**) mRNA was detected by qPCR.** (H)** NICD level decreased in SRCAP-knockout MARC-145 cell line. SRCAP-KO1, SRCAP-KO2, and VEC cells were infected with TA-12. The cellular proteins were collected at 36 HPI and analyzed by Western blot. **(I through M)** Valproic acid or LY-411575 had no effect on PRRSV infection and Notch signaling in the SRCAP-knockout MARC-145 cell line. Valproic acid (Notch signaling activator) or LY-411575 (Notch signaling inhibitor) was added onto SRCAP-KO1, SRCAP-KO2, and VEC cells for 24 h and then were infected with TA-12 at an MOI of 0.01 for 30 h. The viral genome (**I**), mRNA level of HES1 (**J**), HES5 (**K**), and RBP-Jκ (**L**) were detected by qPCR and the viral protein was detected by IFA (**M**).** (N through Q)** Confirming the role of SRCAP in PRRSV life cycle. The replication of the PRRSV was blocked in SRCAP-knockout MARC-145 cell line. In SRCAP-KO1, SRCAP-KO2, and VEC cells, the adsorption (**N**), internalization (**O**), replication (**P**), and release (**Q**) of TA-12 were analyzed using the methods described in Fig. 3. Data were quantified and shown as the ratio of SRCAP to GAPDH. GAPDH was used as an internal control for qPCR. Error bars indicate the SDs of three experimental replicates. GAPDH was used as an internal control for the Western blot. The asterisks indicate a significant difference between the control values: *P < 0.05; **P < 0.01 (Student’s *t* test).

To learn the impact of complete loss of SRCAP, CRISPR/Cas9-mediated SRCAP knockout MARC-145 cells, SRCAP-KO, were developed (Fig. 5A and B). The knockout of SRCAP could decrease PRRSV replication (Fig. 5B through D). The activation of Notch signaling was inhibited after SRCAP knockout (Fig. 5E through H). To further confirm the role of SRCAP in PRRSV-activated Notch signaling, the LY-411575 (inhibitor of Notch signaling) or valproic acid (activator of Notch signaling) was added into the SRCAP-KO cells. The results showed that after knocking out of SRCAP, the LY-411575 or valproic acid had no influence on the expression of HES1, HES5, or RBP-Jκ (Fig. 5I through M). All these results indicated that PRRSV infection activated non-canonical Notch signaling which was dependent on SRCAP. The role of SRCAP in PRRSV life cycle was also detected in the SRCAP-KO cells. The results showed that knockout of SRCAP significantly decreased the replication of the PRRSV (Fig. 5N through Q) while not in other stages. These results were co-incident with those in SRCAP knocked-down MARC-145 cells.

### SRCAP activates non-canonical Notch signaling by interacting with Nsp4 dependently

**Fig 6:**
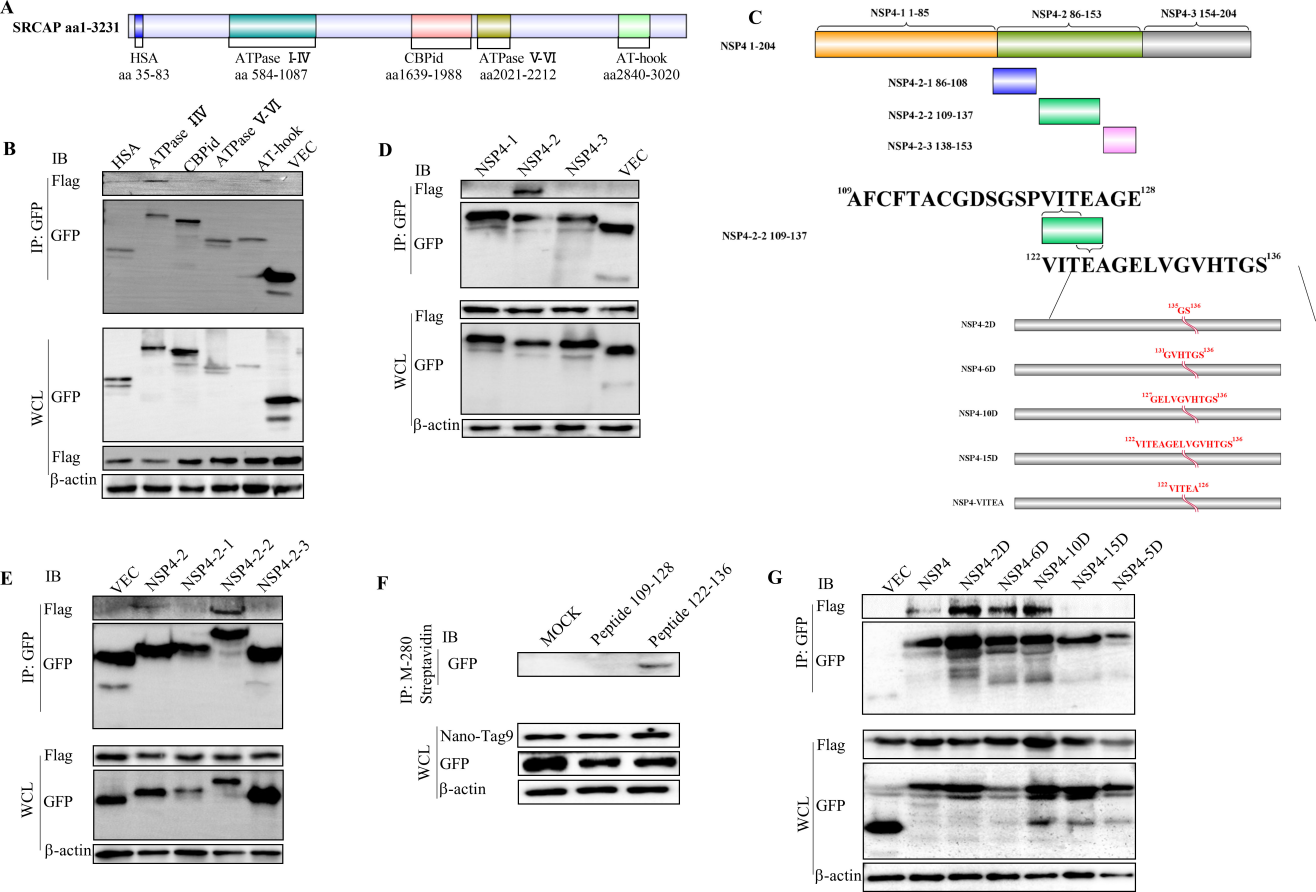
Identification of the interaction region between SRCAP and Nsp4. **(A)** Schematic representation of the structural domains of SRCAP. **(B)** The ATPase I–IV structural domain of SRCAP interacted with Nsp4. Constructs with GFP tags containing five functional domains of HSA, ATPase I–IV, CBPid, ATPase V–VI, and TA-hook were transfected into HEK293T cells. The interacted cellular proteins were pulled down by GFP-Trap and analyzed by Western blot. **(C)** Schematic diagram of truncated Nsp4 proteins. The interacting domain in Nsp4 with ATPase I–IV. Constructs with a GFP tag containing three truncated Nsp4-1, Nsp4-2, Nsp4-3 (**D**) or Nsp4-2, Nsp4-2-1, Nsp4-2-2, and Nsp4-2-3 (**E**) were transfected into HEK293T cells together with Flag-tagged ATPase I–IV. The interacting cellular proteins were pulled down by GFP-Trap and analyzed by Western blot. **(F)** The peptide in Nsp4 interacting with ATPase I–IV. The construct of GFP-tagged ATPase I–IV were transfected into HEK293T cells and the total cellular proteins were collected at 24 h post-transfection. Two synthesized peptides spanning aa 109–128 and aa 122–136 covering Nsp4-2-2 with modification of Biotin-Ahx at the N-terminal were incubated with the cellular proteins, respectively. The peptide-binding proteins were pulled down using Dynabeads M-280 Streptavidin. The isolated cellular proteins were identified by Western blot using Nano-tag, a streptavidin-binding peptide, and an anti-GFP antibody. **(G)** The key sites in Nsp4 with ATPase I–IV. Constructs with a GFP tag containing truncated Nsp4, Nsp4-2D, Nsp4-6D, Nsp4-10D, Nsp4-15D, and Nsp4-5D were transfected into HEK293T cells together with Flag-tagged ATPase I–IV, respectively. The interacting cellular proteins were pulled down by GFP-Trap and analyzed by Western blot. β-actin was used as an internal control for the Western blot.

To find the interacting domain of SRCAP with Nsp4, five functional domains [HAS (aa 35–83), ATPase I–IV (aa 584–1,087), CBPid (aa 1,639–1,988), ATPase V–VI (aa 2,021–2,212), and TA-hook (aa 2,840–3,020)] of SRCAP were constructed and IP was applied (Fig. 6A). The results showed that ATPase I–IV interacted with Nsp4 (Fig. 6B). Nsp4 was also truncated to identify the interacting domain with ATPase I–IV (Fig. 6C). GFP pull-down results showed that Nsp4-2 with amino acid spanning 86–153 interacted with ATPase I–IV (Fig. 6C and D). Nsp4-2 was sub-truncated and GFP pull-down results showed that Nsp4-2-2 with amino acid spanning 109–137 interacted with ATPase I–IV (Fig. 6C and E). To uncover the specific interacting domain, two peptides spanning aa 109–128 and aa 122–136 covering Nsp4-2-2 were synthesized with modification of Biotin-Ahx at the N-terminal (Fig. 6C). The results showed that only Biotin-labeled peptide spanning 122–136 interacted with ATPase I–IV (Fig. 6C and F). To find the key amino acids determining the interaction of ATPase I–IV and Nsp4, five mutant constructs were prepared. The pull-down results showed that only Nsp4 with 15-aa deletion and 5-aa deletions could not interact with ATPase I–IV (Fig. 6C and G). These results indicated that ^122^VITEA^126^ determine the interaction of SRCAP and Nsp4.

**Fig 7:**
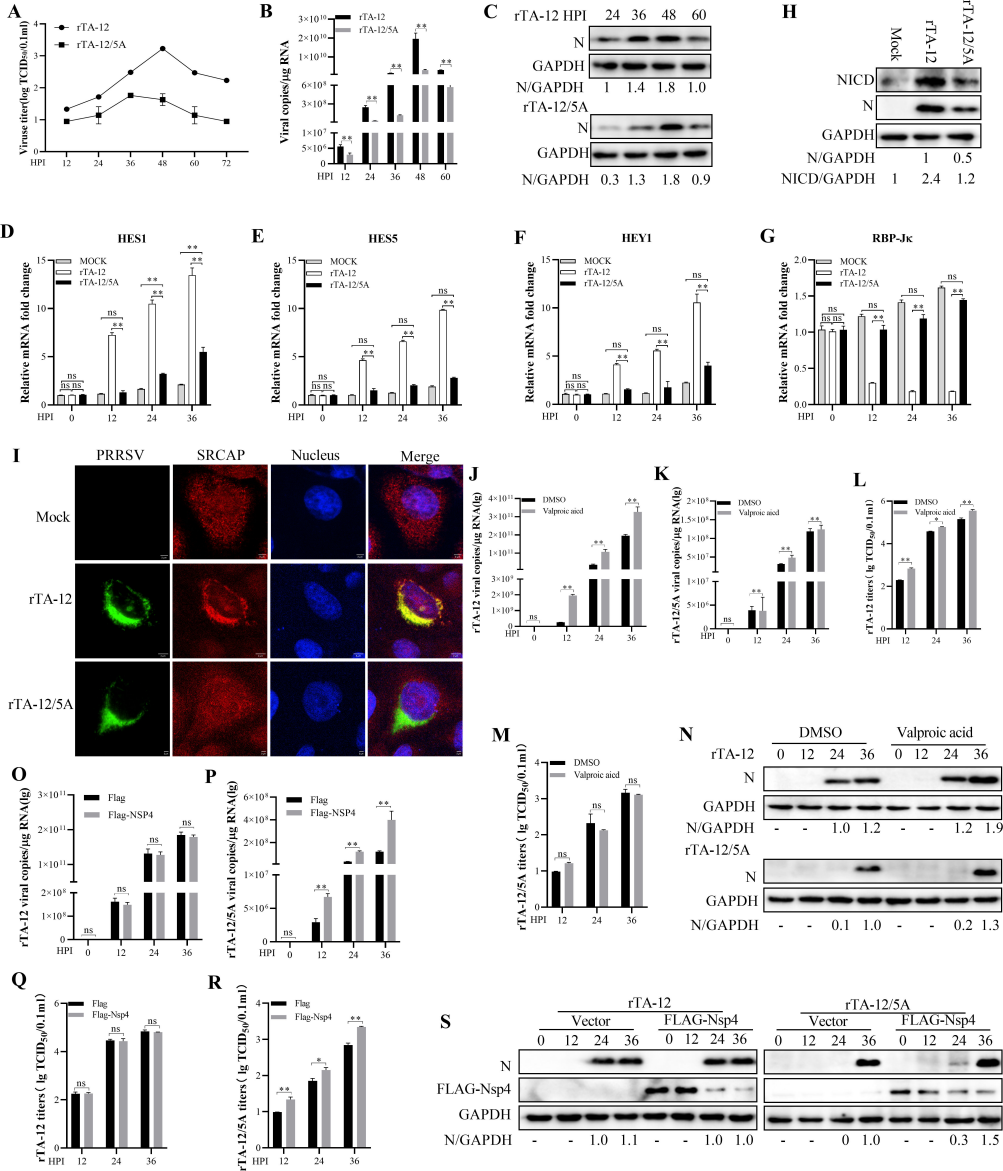
The interaction of Nsp4 with SRCAP is indispensable for the function of SRCAP. **(A)** The multi-step virus growth curves. MARC-145 cells in a 24-well plate were infected with recombinant viruses of passage 2 at an MOI of 0.01, respectively. Virus supernatants harvested at 12, 24, 36, 48, 60, and 72 HPI were titrated by TCID_50_ assay. Each data point represents the mean ± deviation of duplicates. **(B and C)** The replication of rescued PRRSV rTA-12/5A. MARC-145 cells in a 24-well plate were infected with the viruses of passage 2 at an MOI of 0.01, respectively. Both mRNA and protein were collected at 12, 24, 36, 48, and 60 HPI and detected qPCR (**B**) and Western blot (**C**). **(D through G)** The inability of rTA-12/5A on the expression of Notch signaling-related genes. Rescued viruses of rTA-12 and rTA-12/5A of passage 2 were inoculated on MARC-145 cells at an MOI of 0.1 for indicated different time points. The expression of HES1 (**D**), HES5 (**E**), HEY1 (**F**), and RBP-Jκ (**G**) was detected by qPCR.** (H)** rTA-12/5A decreases the nuclear distribution of NICD. MARC-145 cells were infected with recombinant viruses of passage 2 at an MOI of 0.01, respectively. The cellular proteins were collected at 36 HPI and analyzed by Western blot.** (I)** No co-localization of SRCAP with rescued rTA-12/5A. rTA-12- and rTA-12/5A-infected MARC-145 cells were fixed and probed by anti-SRCAP and anti-Nsp4 specific antibodies. The co-localization of Nsp4 with SRCAP was visualized under confocal microscopy. **(J through N)** Valproic acid could not activate Notch signaling after rTA-12/5A infection. Valproic acid, an activator of Notch signaling, was added to MARC-145 cells for 24 h. Then, the rescued viruses of rTA-12- and rTA-12/5A with passage 2 were inoculated on MARC-145 cells at an MOI of 0.1. The viral genome (**J and K**), titer (**L and M**), and protein were detected (**N**). (**O through R**) Nsp4 rescues the replication of rTA-12/5A. MARC-145 cells were transfected with Flag-Nsp4 for 24 h and then infected with the rTA-12 or rTA-12/5A. At 0, 12, 24, 36 HPI the viral genome (**O and P**) and viral titer (**Q and R**) and Western blot (**S**). GAPDH was used as an internal control for qPCR. The error bars indicate the SDs of three experimental replicates. GAPDH was used as an internal control for the Western blot. The asterisks indicate a significant difference between the control values: *P < 0.05; **P < 0.01 (Student’s *t* test).

To further confirm the role of the interaction between SRCAP and Nsp4, a mutant PRRSV (rTA-12/5A) based on TA-12 (GenBank no. HQ416720) with ^122^AAAAA^126^ in Nsp4 was recovered. The recovered rTA-12/5A showed low viral titers and replication efficiency compared with rTA-12 (Fig. 7A through C). No changes on the HES1, HES5, HEY1, and RBP-Jκ were found in rTA-12/5A infected cells (Fig. 7D through G). Compared with rTA-12, rTA-12/5A infection did not affect the protein level of NICD (Fig. 7H). The co-localization of the Nsp4 and SRCAP was found in rTA-12 infected MARC-145 cells while not found in those of rTA-12/5A (Fig. 7I). All these results indicated that SRCAP was indispensable for the PRRSV-activated Notch signaling pathway. Valproic acid, an activator of Notch signaling pathway, was used to learn the role of Notch signaling pathway in PRRSV infection. The results showed that valproic acid could increase the replication of rTA-12 not rTA-12/5A (Fig. 7J through N). To further confirm the role of the key sites of Nsp4 in PRRSV infection. MARC-145 cells were transfected with FLAG-Nsp4 or black vector and then infected with rTA-12 or rTA-12/5A. The results showed that transfection of FLAG-Nsp4 could increase the replication of the rTA-12/5A not rTA-12(Fig. 7O through S). All these results indicated that the activation of SRCAP on non-canonical Notch signaling is dependent on its interaction with Nsp4. The ^122^VITEA^126^ in Nsp4 were key sites to determine the function of SRCAP and their interaction.

### Block Notch signaling pathway inhibits PRRSV infection *in vitro* and *in vivo*

**Fig 8:**
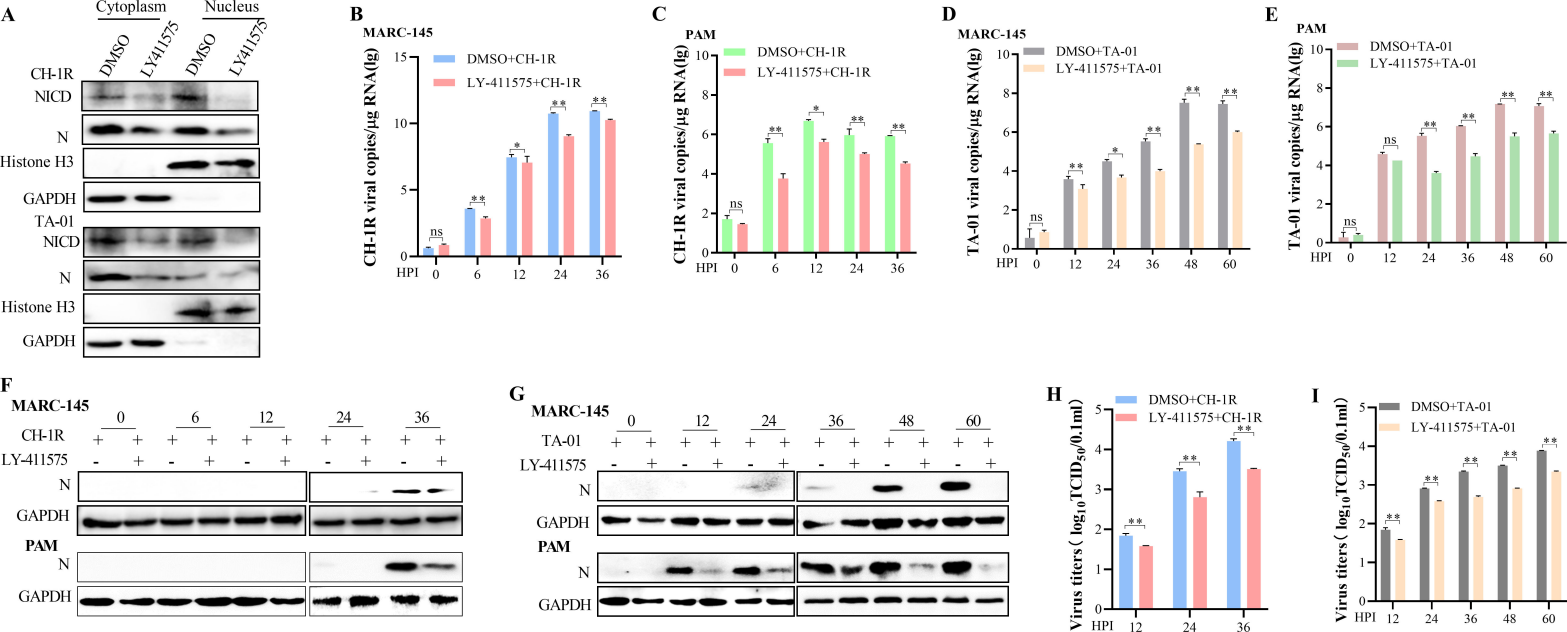
Notch signaling pathway inhibitor, LY-411575, could inhibit PRRSV infection *in vitro*. **(A)** LY-411575 altered the distribution of NICD after PRRSV infection. LY-411575 was added to the MARC-145 cells for 24 h and then infected PRRSV strains of CH-1R or TA-01 for 24 h. Cytoplasmic protein and nucleus protein were prepared and detected by Western blot. **(B through I)** LY-411575 decreases the PRRSV genome. LY-411575 was added to the MARC-145 cells or PAMs for 24 h followed by an inoculation with different strains of PRRSV. Total RNA was collected at 0, 6, 12, 24, and 36 h. The viral genome of CH-1R was evaluated in MARC-145 cells (**B**) and PAMs (**C**). The viral genome of TA-01 was evaluated in MARC-145 cells (**D**) and PAMs (**E**). The viral proteins were detected by Western blot (**F and G**). The viruses in the supernatant were titrated (**H and I**). GAPDH was used as an internal control for qPCR and Western blot. The error bars indicate the SDs of three experimental replicates. GAPDH was used as an internal control for the Western blot. The asterisks indicate a significant difference between the control values: *P < 0.05; **P < 0.01 (Student’s *t* test).

The above results showed that PRRSV infection activated non-canonical Notch signaling pathway to facilitate its replication and the inhibitor of this pathway (LY-411575) could block TA-12 infection. To study the spectrum of antiviral role of LY-411575, PRRSV strain CH-1R and TA-01 were detected. First, the effects of LY-411575 on Notch signaling pathway were first confirmed in the CH-1R and TA-01-infected MARC-145 cells. The results showed that the nuclear NCID levels decreased after adding LY-411575 (Fig. 8A). LY-411575 could decrease the viral genome (Fig. 8B through E), protein (Fig. 8F and G), and viral titration (Fig. 8H and I) of CH-1R and TA-01 significantly in both types of cells. All these results indicated that LY-411575 was an effective inhibitor of a wide types of PRRSV infection by inhibiting the Notch signaling pathway.

**Fig 9:**
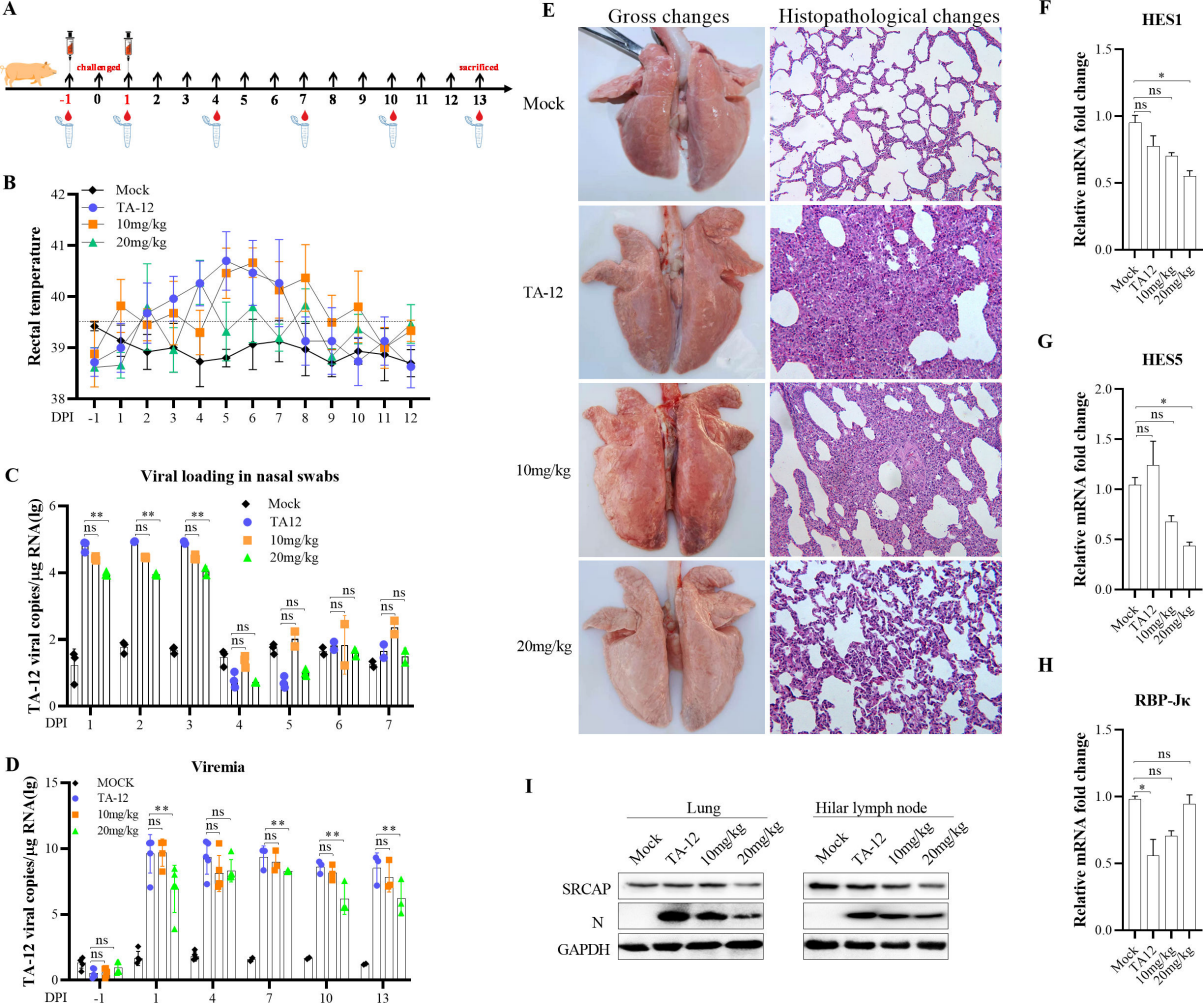
LY-411575 inhibited PRRSV infection *in vivo.* (**A**) Pattern diagram of animal experiments. (**B**) Rectal temperature of piglets from each group presented as the mean ± SD (error bars). A temperature ≥39.5°C was defined as a clinical fever. (**C**) Viral RNA load in the nasal swabs (log copies*mL^−1^). (**D**) Viral load in the sera of pigs (log copies*mL^−1^). (**E**) Gross and microscopic observations of the lungs collected from the piglets. (**F through H**) Effect of LY-411575 on Notch signaling activated by PRRSV *in vivo*. The level of *HES1* (**F**), *HES5* (**G**), and *RBP-Jκ* (**H**) mRNA expression in the lungs at day 5 were evaluated by qPCR. (**I**) Protein in the lungs and hilar lymph node at day 5 were detected by Western blot. GAPDH was used as an internal control for qPCR and Western blot. Error bars indicate the SDs of three experimental replicates. GAPDH was used as an internal control for the Western blot. The asterisks indicate a significant difference between the control values: *P < 0.05; **P < 0.01 (Student’s *t* test).

To determine the effects of LY-411575 on PRRSV infection *in vivo*, TA-12 was inoculated into pigs following an administration of LY-411575 by intramuscular injection, and the samples were collected at the indicated times (Fig. 9A). The results showed that LY-411575 could reduce the elevated body temperature, viremia, and viral load in the nasal swabs caused by infection with TA-12 (Fig. 9B through D). The gross and histopathological changes of the lung were also alleviated by LY-411575 (Fig. 9E). Further study showed that viral infection upregulated the level of HES1 and HES5 mRNA expression and downregulated that of RBP-Jκ. LY-411575 could further downregulate the level of HES1 and HES5 mRNA expression and restore RBP-Jκ expression caused by TA-12 infection (Fig. 9F through H). In both the lungs and hilar lymph nodes, infection with TA-12 could increase the level of SRCAP, whereas the addition of LY-411575 could reduce the viral load (Fig. 9I). All these findings indicate that TA-12 infection could activate Notch signaling *in vitro*, which could be blocked by LY-411575. LY-411575 may function as an effective drug to relieve TA-12 infection by inhibiting activation of the Notch signaling pathway.

## DISCUSSION

PRRS, a problematic disease for the swine industry worldwide because, at present, there are no effective vaccines or drugs against the PRRSV. Uncovering the influence on cellular biology during PRRSV infection is very important to contain PRRS. As a classical and fundamental signaling pathway, the aberration of Notch signaling leads to various disorders and outcomes during virus infection (27–29). In the canonical Notch signaling pathway, RBP-Jκ is constitutively expressed and binds with NICD to regulate the transcription of Notch target genes, whereas non-canonical Notch signaling is RBP-Jκ-independent (8, 30–32). Notch signaling has been found to contribute to the expression of inflammatory cytokines during PRRSV infection (12). In this study, the expression of RBP-Jκ decreased during PRRSV infection which indicated that non-canonical Notch signaling was involved in PRRSV infection.

PRRSV infection activates non-canonical Notch signaling to facilitate its replication. SRCAP played key role in PRRSV inducing Notch signaling pathway by interacting with Nsp4. The activator or inhibit of Notch signaling could not change the expression of target genes in SRCAP-knocked out MARC-145 cells (Fig. 5). These results further confirmed that PRRSV-activated Notch signaling was SRCAP-dependent. However, the expression of HES1 and HES5 *in vitro* and *in vivo* was different (Fig. 4 and 9). *In vitro* study, the expression of HES1 and HES5 was detected in one cell type of MARC-145. While *in vivo* study, Hes1 and hes5 expressions of multiple cells were detected. Most cell types were not susceptible for PRRSV, which may be the reason for the non-significant expression changes of HES1 and HES5. ATPase I–IV domain in SRCAP interacted with the motif of ^122^VITEA^126^ in Nsp4 was found in this study. The interaction is indispensable for the function of SRCAP in Notch signaling. Based on these results, the mutant virus with ^122^AAAAA^126^ (rTA-12/5A) was rescued which may be a candidate for PRRSV vaccine. Unfortunately, the recovered rTA-12/5A showed lower titer compared with wild-type rTA-12 and cannot be detected after passing for five passages. All these results indicated that the motif of ^122^VITEA^126^ in Nsp4 is prerequisite for successful infection of PRRSV.

Like other positive-stranded RNA viruses, viral non-structural proteins were presumed to be part of the replication/transcription complex (RTC) in which replication of the viral genome and transcription of viral mRNA occurred (23, 33–35). Although the role of PRRSV Nsp4 in the RTC remains unknown, similar Nsp4s were reported to be important in severe acute respiratory syndrome coronavirus and murine hepatitis virus; both of which share similar genomic structures and transcriptional strategies with PRRSV (35). In the present study, the biological processes and molecular functions of Nsp4/protein interactions were mainly related to RNA binding and gene expression; thus, confirming the role of Nsp4 in regulating RNA transcription. It was reported that Nsp4 bound to the porcine β2-microglobulin gene (*B2M*) promoter and inhibited its transcription via interactions between Nsp4 and the enhancer *PAM* element of the porcine *B2M* promoter (36). In the current study, SRCAP, a high-molecular-weight protein (350 kDa) member of the SNF2 protein family of DNA-dependent ATPases, was deemed the most likely interacting partner for Nsp4. SCRAP is known to play roles in chromatin remodeling, DNA repair, transcript regulation, and viral infection (37). In particular, it was found that SRCAP aided in the transcriptional activation of the hepatitis C viral protein, NS5A (15). A previous study indicated that adenovirus 2 DNA binding protein inhibited transcriptional activation by binding to the carboxyl-terminal region of SRCAP (18, 19). In agreement, we found that Nsp4 pulled down SRCAP, and also SRCAP could be detected in GFP-Trap products. Knocking down of SRCAP also significantly inhibited PRRSV infection. Taken together, we postulate that SRCAP may promote viral infection by activating viral RNA transcription, although further study is needed to confirm the hypothesis.

The diverse role of Notch signaling pathway in pathological conditions has drawn attention to pharmacological interventions. The main strategies include disrupting the proteolytic cleavage/processing of Notch or inhibition of Notch−ligand interactions. Currently, many drugs with Notch-targeting therapeutic strategies have been searched and are in the preclinical stage or tested in clinical trials, especially in treating cancer, immunity, and inflammatory disorders (38–41). The use of GSIs has been the most explored way of controlling Notch activation (42). More than 100 GSIs have been designed, synthesized, and evaluated (43). LY-411575 is a potent γ-secretase inhibitor and also inhibits Notch S3 cleavage effectively (44, 45). LY-411575 is one member of azepines with the function of non-competitive substrate docking site binders (43). So, in this study, LY-411575 was selected as a GSI to inhibit Notch signaling both *in vitro* and *in vivo*. LY-411575 was confirmed effective in blocking PRRSV infection, which supplied a potential candidate drug containing PRRS.

In conclusion, SRCAP was identified as one of the interactors for Nsp4 and acted as a proviral host factor for PRRSV replication. SRCAP facilitated PRRSV infection by activating non-canonical Notch signaling which was determined by the motif of ^122^VITEA^126^ in Nsp4. A function of Nsp4 in activating Notch signaling pathway was discovered. Block Notch signaling pathway could inhibit PRRSV infection *in vitro* and *in vivo* which could be used as potential antiviral therapies.

## MATERIALS AND METHODS

### Cells and virus

MARC-145 and 293T cells were from the China Center for Type Culture Collection (CCTCC, Wuhan, China) and were cultured in Dulbecco’s modified Eagle’s medium (DMEM) (Gibco, Langley, OK, USA) supplemented with 10% fetal bovine serum at 37°C, 5% CO_2_ in a humidified incubator. The low-pathogenic PRRSV strain CH-1R and PK15^CD163^ (PK15 cell line stably expressing CD163) cells were kindly provided by Dr. Qin Zhao (Northwest A&F University, China). The HP-PRRSV strain TA-12 (GenBank no. HQ416720), NADC30-like strain TA-01, and recombinant strain (recombined by HP-PRRSV and NADC30-like) strain TA-02 were previously isolated and stored in our lab. PAMs were isolated from five healthy 5-week-old crossbred weaned pigs (Landrace × Yorkshire) as previously described (46).

### Plasmids

The pEGFP-C1 vector (Clontech Laboratories, Inc., Mountain View, CA, USA), pCMV-Myc (Clontech Laboratories, Inc., Mountain View, CA, USA), and p3XFLAG-myc-CM-26 (Sigma-Aldrich, USA) were used to create expression constructs. The details on the constructs and primers were listed in Table 2.

**TABLE 2:** Information on synthesized gene sequences

Vector	Gene	Forward (5’−3’)	Reverse (5’−3’)
Primers for constructs			
pEGFP-C1	Nsp4	ACAAGCTTCGGGTGCTTTCAGAACTCAA	AGTCGACTTATTCCAGT TCGGGTTTGGCAGCA
	Nsp4-1	GTCGACGGTACCGCGGGCATGGGTGCTTTCAGAACTC	TGCAGAATTCGAAGCTTGAGCTTAATCCTCGCAGAACTGGGC
	Nsp4-2	GTCGACGGTACCGCGGGCATGGGGTGGACTGGTCGC	TGCAGAATTCGAAGCTTGAGCTTAACAAAACTGGCCTGAG
	Nsp4-3	GTCGACGGTACCGCGGGCATGAATGTGAAGCCCATC	TGCAGAATTCGAAGCTTGAGCTTATTCCAGTTCGGGTTTG
	Nsp4-2-1	GTCGACGGTACCGCGGGCATGGGGTGGACTGGTCGCGC	TGCAGAATTCGAAGCTTGAGCTTAGAACCCATTCCCAATAAC
	Nsp4-2-2	GTCGACGGTACCGCGGGCATGGCCTTTTGCTTCACCGC	TGCAGAATTCGAAGCTTGAGCTTATGATCCTGTGTGAACGCC
	Nsp4-2-3	GTCGACGGTACCGCGGGCATGAACAAACAAGGAGGAG	TGCAGAATTCGAAGCTTGAGCTTAATTACAAAACTGGCCT
	Nsp4-2D	CGGCGTTCACACAAACAAACAAGGAGGAGGCATTG	TTGTTTGTGTGAACGCCGACAAGCTCACCG
	Nsp4-6D	CGGTGAGCTTGTCAACAAACAAGGAGGAGGCATTG	TTGTTGACAAGCTCACCGGCTTCGGTAATCACT
	Nsp4-10D	CCGAAGCCAACAAACAAGGAGGAGGCATTGTCA	CTTGTTTGTTGGCTTCGGTAATCACTGGGGATC
	Nsp4-15D	CCCCAGTGATTACCAACAAACAAGGAGGAGGCATTG	TGTTGGTAATCACTGGGGATCCAGAATCGCCAC
	Nsp4-5D	ATCCCCAGGTGAGCTTGTCGGCGTTCACACAGG	ACAAGCTCACCTGGGGATCCAGAATCGCCACAC
	HSA	GAAGATCTATGCAGAGCAGCCCCTCCCC	GGGGTACCTTACTGAGACAGAAGGTCCGAGT
	ATPase I-IV	GAAGATCTATTACTGACATTGCTGC	GGGGTACCTTAGAGAGAACCACTGTTGGGC
	CBPid	GGAATTCTGGAACCTCTTTAGCCTCAGC	CGGGATCCTTAGTGCGCATGTTACACAC
	ATPase V-VI	GAGGTACCATGTCCCTGCATGCCTGCCACCC	TTGGATCCTTAGCCACCACCAGTCACCCCC
	TA-hook	GAAGATCTAGCCCCGAGAATGGAGAC	GGGGTACCTTAGCTGGGCCGAGGTGATGGAG
PcDNA3.0 FLAG	Nsp4	GGGGTACCGGTGCTTTCAGAACTCAA	GCTCTAGACTTTATTCCAGTTCGGGTTTGGC
qPCR primers			
Monkey genes	Gene	Sequence 5′−3′	Antisense 5'−3'
	GAPDH	ACCCACTCTTCCACCTTCGACGCT	TGTTGTTGTAGCCAAATTCG
	srcap	ACTTCATGGCACAAACCACA	GAGGGTCGAACAGATTTGGA
	hes1	AGAATAAATGAAAGTCTGAGCC	TCGTTCATGCACTCGCTG
	hes5	CGTTGCCTCCTCGCCTGTTCC	ATGTCGGCCTTCTCCAGC
	rbp-jκ	GATTCAGACAAGCGAAAGCAC	TCGATTAAACAGAGCCACC
Swine genes	GAPDH	CCTTCCGTGTCCCTACTGCCAAC	GACGCCTGCTTCACCACCTTCT
	srcap	CGGAAACAGCAGCGGTC	CGAGGGCCGATGGGGCTGGC
	hes1	CGACACCGGATAAACCAAAGACAG	CGCGAGCTATCTTTCTTCAGAGCA
	hey1	GCGACCGCATCAACAGCA	CTGTAGTCCTGGTGCAG
	hes5	GCTGACTTATGCATTGCCTCAGGA	ACTCATCATCATCCAAG
	rbp-jκ	GCAGACTCCACTCCACTT	GGACTCCCACACTGGCCAG
PRRSV	N	AGATCATCGCCCAACTAAAC	GACACAATTGCCGCTCACTA
siRNA primers			
Monkey genes	Nucleate site	Sequence 5′−3′	Antisense 5'−3'
NC		UUCUCCGAACGUGUCACGUTT	ACGUGACACGUUCGGAGAATT
SRCAP	1285	GGAAACGAUUGAAGUUGAATT	UUCAACUUCAAUCGUUUCCTT
	1694	GCUCGAGAAGGUGAGCUUUTT	AAAGCUCACCUUCUCGAGCTT
	2108	CCCACUACUCUAGGUCCAATT	UUGGACCUAGAGUAGUGGGTT
Swine genes			
SRCAP	756	GCUGUUGAGAUCCUGCCUATT	UAGGCAGGAUCUCAACAGCTT
	1736	CCACCACUCUAGGUCCUAATT	UUAGGACCUAGAGUGGUGGTT

### Transfection

For pull-down immunoprecipitation assays, 293T cells were seeded and cultured to 50% confluence prior to plasmid transfection using X-tremeGENE HP DNA transfection reagent (Roche, Shanghai, China) according to manufacturer’s instructions. Monolayer MARC-145 cells were transfected with Lipofectamine 2000 (Sigma-Aldrich, St Louis, MO, USA) using a protocol adapted from the immunofluorescence (IF) assay (see below).

Monolayer MARC-145 cells were transfected with either 25 µM of gene-specific small interfering (si) RNAs targeting SRCAP or control siRNA (Table 2; GenePharma, Shanghai, China) using Lipofectamine RNAiMAX (Invitrogen) according to the manufacturer’s instructions. Twenty-four hours later, the cells were inoculated with PRRSV TA-12 at 0.01 multiplicity of infection (MOI); the cells were collected at 0, 6, 12, 24, and 36 h post-infection (HPI) and the protein and RNA levels were detected by Western blotting and quantitative real-time (qRT) PCR assays.

### Antibodies and reagents

Antibodies of anti-MYH10 (Santa Cruz Biotechnology, CA, USA), anti-SRCAP (Santa Cruz Biotechnology), and anti-TRIM28 (Cell Signaling Technology, Danvers, USA) were used in this research. An anti-SRCAP antibody (sc-133310) was purchased from Santa Cruz. Anti-β-actin antibody (AP0060) was obtained from Bioworld Technology. Anti-GAPDH (AT1749), anti-Flag (AT0022), and anti-GFP (AT0028) antibodies were purchased from Engibody Biotechnology. Anti-NICD (4147), antibody was obtained from Cell Signaling Technology. Anti-His (66005-1-Ig) antibody was acquired from Proteintech. Anti-Nano-tag9 (A02300) antibody was obtained from Amyjet Scientific. Anti-TRIM28 (ab109287) antibody was purchased from Abcam. LY-411575 (HY-50752) and valproic acid (HY-10585) were acquired from MedChemExpress. Monoclonal antibodies against the PRRSV nucleocapsid and Nsp4 were prepared in our lab (46).

### GFP pull-down, Co-IP assay, and peptide pull-down

For the GFP pull-down assays, monolayers of HEK293T cells in 10 cm dishes were transfected with recombinant plasmids for 24 h. The cells were harvested and lysed. Clarified cellular lysates were incubated with GFP-Trap beads (Chromotek, Germany) for 2 h at 4°C, and the bound proteins were eluted with 100 µL of 2 ×  SDS sample buffer. For the Co-IP assays, cell lysates were prepared and incubated with anti-Flag, SRCAP, and TRIM28 antibodies, respectively. Protein G resin was added and incubated for 2 h. The bound proteins were eluted with sample buffer after extensive washing and loaded onto an SDS-PAGE gel for immunoblotting analysis.

To identify the interaction domain in Nsp4-2-2 with SRCAP-8, the GFP-tagged SRCAP-8 construct was transfected into HEK293T cells and the total cellular proteins were collected after 24 h. Two synthesized peptides spanning aa109-128 and aa122-136 covering Nsp4-2-2 with a modification of Biotin-Ahx at the N-terminal were incubated with the cellular proteins, respectively. The peptide-binding proteins were pulled down using Dynabeads M-280 Streptavidin and analyzed by Western blot using Nano-tag, a streptavidin-binding peptide, and an anti-GFP antibody.

### LC-MS/MS assay

Monolayers of HEK293T cells in 10 cm dishes were transfected with recombinant plasmids (three dishes/plasmid). At 24 hpt, the cells were harvested and lysed. Clarified cell lysates were incubated with GFP-Trap beads (Chromotek, Germany) for 2 h at 4°C, and the bound proteins were eluted with 100 µL of 2 ×  SDS sample buffer, or the bound proteins were added 0.2 M glycine, incubated for 10 min and then the supernatant was centrifuged, 1 M tris base was added to balance the acidity and the obtained samples were analyzed by LC-MS/MS analysis as described previously (26). Briefly, LC-MS/MS analysis was performed on a Q Exactive mass spectrometer (operated in positive ion mode; Thermo Scientific) that was coupled to Easy nLC (Thermo Fisher Scientific). MS data were acquired using a data-dependent top 10 method that dynamically chose the most abundant precursor ions from the survey scan (300–1800 m/z). Survey scans were acquired at a resolution of 70,000 at 200 m/z; the resolution for MS^2^ scan (HCD) spectra was set to 17,500 at 200 *m/z*; and the isolation width was 2 *m/z*. The MS data were analyzed using MaxQuant software version 1.3.0.5 (Max Planck Institute of Biochemistry, Martinsried, Germany). The false discovery rates were set at 1% and at least two unique peptides were required for reporting protein identifications.

### Western blotting

The samples were separated using 8–15% SDS-PAGE and transferred to polyvinylidene difluoride membrane membranes (Millipore, MA, USA) using a Bio-Rad semi-dry transfer apparatus according to standard procedures. Membranes were probed with the primary antibody, anti-GFP (CMCTAG, Shanghai, China), as well as all antibodies mentioned above. Horseradish peroxidase-conjugated anti-mouse or anti-rabbit secondary antibodies were then added. Blots were imaged using the Clarity Western ECL kit (Bio-Rad).

### Confocal microscopy

293T cells were seeded onto coverslips in 24-well plates and transfected with plasmids using Lipofectamine 3000 reagent (Invitrogen, USA). MARC-145 cells were either mock-infected or infected with PRRSV at an MOI of 0.01. After 24 h, the cells were fixed with 4% paraformaldehyde (Beyotime Biotech, China) for 15 min, permeabilized with 0.1% (vol/vol) triton X-100 (Solarbio, China) in phosphate-buffered saline (PBS). After blocking with 1% bovine serum albumin (BSA; Solarbio, China) in PBS for 1 h, the cells were incubated with primary antibodies for 2 h. Cells were then washed and incubated with fluorescently labeled secondary antibodies conjugated to fluorescein isothiocyanate (FITC) or Cyanine 3 (Cy3). Cell nuclei were stained with 4′,6-diamidino-2-phenylindole (DAPI; Invitrogen, USA). All probed cells were observed under a fluorescence microscope (Leica, SPE, Buffalo Grove, IL, USA).

### Rescue of recombinant PRRSV

Plasmid pACYC177-CMV-MCS-HDV-SV40, which contains a cytomegalovirus (CMV) promoter, a multiple-cloning site (MCS), a hepatitis delta virus (HDV) ribozyme, and a simian virus 40 (SV40) signal, was modified from the low-copy-number plasmid vector pACYC177 used previously for constructing PRRSV infectious clones. Briefly, the full-length cDNA clone of HP-PRRSV TA-12 or mutation of ^122^AAAAA^126^ of Nsp4 gene was assembled by homologous recombination. The recombinant wild-type (rTA-12) or mutant (TA-12Nsp4-5A) viruses were recovered by transfecting BHK-21 cells. The titration of viruses was measured by TCID_50_ and viral growth curves were created with GraphPad Prism 8.

### qRT-PCR analysis

RNA was extracted using ReverTra Ace qPCR RT Kit (TOYOBO, Japan) according to the manufacturer’s instructions and qRT-PCR was performed using specific primers (Table 2). GAPDH was used as an internal control. The PRRSV genome was detected by the Nucleocapsid (N) gene.

### Generation of SRCAP knockout cells

A CRISPR-Cas9 vector system carrying two guide RNA-expressing cassettes was used to construct recombinant plasmids: (i) target 1 (CAS9SRCAPF1: CACCGTTTACTGCGCCGA GTTAAGGTGG; CAS9SRCAPR1: AAACCCTTAACTCGGCGCAGTAAAC), which was inserted into PX459M; and (ii) target 2 (CAS9SRCAPF2: CACCGGCCTTTTTACTGCGCC GGTGGG; CAS9SRCAPR2: AAACACCGGCGCAGTAAAAAGGCC), which was inserted into EZ-GuideXH. Target 1 and target 2 were ligated together to form the CRISPR/Cas9 KO plasmid. The identified recombinant CRISPR/Cas9 KO plasmid was transfected into MARC-145 cells and subsequently sorted by replacing selective media with 10 µg/mL of puromycin (TAKARA, Beijing, China) approximately every 1–2 days for a minimum of 3–5 days. The knockout cells were then confirmed by Western blot and qPCR and termed Marc145^SRCAP-KO^ cells. The blank vectors were also treated with the same procedure and the corresponding cell line was termed Marc145^VEC^ cells.

### PRRSV absorption, internalization, replication, and release assays

The methods for PRRSV absorption and internalization were performed as described previously (47). For PRRSV replication assay, SRCAP knocked down MARC-145 cells or monolayers of SRCAP-KO, VEC cells were inoculated with TA-12 at an MOI of 0.01 at 37°C. At 12,18, and 24 HPI, the culture supernatants infectious virus particles were determined by the TCID_50_. For viral release assay, SRCAP-KO, VEC cells were inoculated with TA-12 at an MOI of 0.01 at 37°C to achieve normal virus replication. The intracellular and extracellular infectious virus particles were determined by the TCID_50_ assay using the freeze-and-thaw cell supernatants and culture supernatants, respectively.

### Animal challenge

A total of 20 five-week-old piglets (free of PRRSV, porcine circovirus type 2, classical swine fever virus, pseudorabies virus, swine influenza virus, and *Mycoplasma hyopneumoniae*) were randomly allocated to four groups (five piglets per group): (i) an uninfected and untreated mock group; (ii) PRRSV-infected and vehicle (PBS + 0.5% DMSO)-treated group; (iii) PRRSV-infected and LY-411575 (10 mg/kg)-treated group; or (iv) PRRSV-infected and LY-411575 (20 mg/kg)-treated group. Piglets were challenged intranasally (1 mL) and intramuscularly (1 mL, in the right neck) with PRRSV strain TA-12 (5 × 10^5^ TCID_50_) or PBS. The piglets were intramuscularly injected in the left neck with 10 or 20 mg/kg of body weight of LY-411575 or 0.5% DMSO in PBS at 24 h before and after infection (Fig. 8A). All piglets were monitored daily for general health status and rectal temperature. Serum samples and nasal swabs were collected at −1, 1, 4, 7, 10, and 13 dpi. All piglets were sacrificed at day 13 dpi and tissues were separated for histopathological observation, Western blot, and qRT-PCR.

### Hematoxylin-eosin (H&E) staining

Tissues were fixed in 4% neutral buffered formalin (Solarbio, China) for 18 h. After dehydration and embedding in paraffin, the tissue samples were cut into 3-μm-thick sections and mounted onto glass slides. For histopathology, the sections were stained with H&E (Thermo Fisher Scientific, USA) according to the manufacturer’s instructions.

### Statistical analysis

Statistical analyses were performed using a one-way analysis of variance when more than two groups were being compared and Student’s *t* test was used when only two groups were compared. The analyses were performed using the SPSS 20.0 software package (version 20.0; SPSS Inc., USA). The data were expressed as the means and standard deviations (SDs) from at least three independent experiments. A *P* value of <0.05 was considered statistically significant.
